# Y chromosome sequencing data suggest dual paths of haplogroup N1a1 into Finland

**DOI:** 10.1038/s41431-024-01707-7

**Published:** 2024-10-28

**Authors:** Annina Preussner, Jaakko Leinonen, Juha Riikonen, Matti Pirinen, Taru Tukiainen

**Affiliations:** 1 Institute for Molecular Medicine Finland (FIMM), HiLIFE, University of Helsinki, Helsinki, Finland; 2 Department of Public Health, Faculty of Medicine, University of Helsinki, Helsinki, Finland; 3 Department of Mathematics and Statistics, University of Helsinki, Helsinki, Finland

**Keywords:** Population genetics, Haplotypes

## Abstract

The paternally inherited Y chromosome is highly informative of genetic ancestry, therefore making it useful in studies of population history. In Finland, two Y-chromosomal haplogroups reveal the major substructure of the population: N1a1 enriched in the northeast and I1a in the southwest, suggested to reflect eastern and western ancestry contributions to the population. Yet, beyond these major Y-chromosomal lineages, the distribution of finer-scale Y-chromosomal variation has not been assessed in Finland. Here, we provide the most comprehensive Y-chromosomal study among the Finns to date, exploiting sequences for 1802 geographically mapped Finnish Y chromosomes from the FINRISK project. We assessed the distribution of common Y-chromosomal haplogroups (frequency ≥1%) throughout 19 Finnish regions and compared the autosomal genetic backgrounds of the Y-chromosomal haplogroups. With such high-resolution data, we were able to find previously unreported sublineages and resolve phylogenetic relationships within haplogroups N1a1 (64%), I1a (25%), R1a (4.3%), and R1b (4.8%). We further find novel geographical enrichment patterns among these Y-chromosomal haplogroups, most notably observed for haplogroup N1a1 dividing into two lineages with differing distributions. While sublineage N-Z1934 (42%) followed a northeastern enrichment pattern observed for all N1a1 carriers in general, sublineage N-VL29 (22%) displayed an enrichment in the southwest. Further, the carriers of N-VL29 showed a higher proportion of southwestern autosomal ancestry compared to carriers of N-Z1934. Collectively, these results point to distinct demographics within haplogroup N1a1, possibly induced by two distinct arrival routes into Finland. Overall, our study suggests a more complex genetic population history for Finns than previously proposed.

## Introduction

**Fig. 1:**
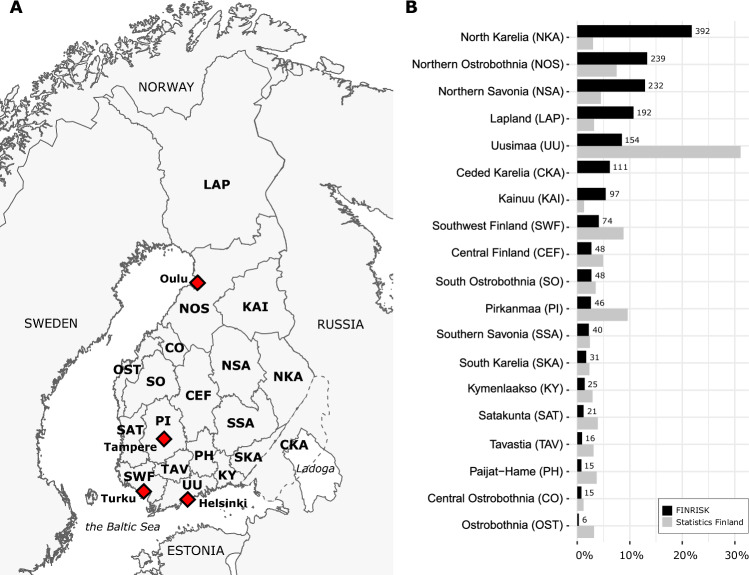
Geographical regions covered in this study. **A** Location of 19 regions used in this study. Red diamonds highlight 4 of the largest metropolitan areas in Finland. The metropolitan area of Helsinki also includes cities of Espoo and Vantaa. **B** Full names of the studied regions and sample sizes based on the assigned geographical locations. Gray bars indicate the current population sizes within each region from Statistics Finland [21], while black bars indicate the FINRISK data distribution. Our data contains excess samples from eastern parts of Finland due to the sampling strategy of the FINRISK project.

Data collected from the Finnish population has been widely used in many genetic studies, ranging from investigations of disease susceptibility to population genetics [1, 2]. Relative isolation within the Northeastern corner of Europe (Fig. 1A), together with small founder populations and several population bottlenecks, have shaped the genetic background of modern Finns distinct from that of other Europeans [3]. Additionally, the Finnish population has been shaped by cultural, political, and linguistic influences, which have led to genetic differences within the country, most notably observed between eastern and western Finland [4–7]. These genetic differences, in part illustrated by distribution of Y-chromosomal haplogroups, are suggested to reflect two separate influences from the eastern and western directions [4, 7, 8].

The majority of Finnish men carry Y-chromosomal haplogroup N1a1 (also known as N1c1 [9], N3 [7, 10]), having an estimated frequency of 58% in the country [7]. N1a1 represents one of Northeast Eurasia’s prominent patrilineages and is enriched among Finno-Ugric-speaking populations [10]. Within Finland, the highest frequencies of N1a1 are observed in the eastern regions of the country [7, 9]. This aligns with the postulated Siberian origin of the haplogroup [11, 12]. Alongside N1a1, a notable proportion of Finnish Y chromosomes belongs to haplogroup I1a, carried in total by 28% of men [7]. While I1a is globally enriched in Scandinavia reaching its peak frequency of 37% in Sweden [13], unlike N1a1, it is also commonly observed across many European countries [14]. In Finland, I1a is especially frequent along the western coast of the country [7]. In addition to these two major Y-chromosomal lineages, ~10% of Finnish men carry haplogroups R1a and R1b, associated with Eastern and Western European ancestries, respectively [7, 15].

While previous Y-chromosomal studies in Finland have provided insights into the major substructure of the population [7, 8], these studies have been limited by the assessment of only a few targeted Y-chromosomal haplogroups. Recent Y-chromosomal studies have demonstrated the power of leveraging the combination of sequencing and genotyping data to discover substructure within major haplogroups [10]. For instance, Ilumäe et al. [10], in their comprehensive assessment of haplogroup N1a1 across Northern Eurasia, showed that among northeastern European populations N1a1 divides into sublineages, N-VL29 and N-Z1936, that have become widespread over the region within the last 5000 years [10]. Further assessment of such finer level variation within individual populations could provide better detail into demographics and elaborate on a population’s history.

In this study, we characterized the phylogeographic landscape of Y chromosome variation in Finland by utilizing sequencing data of 1802 Y chromosomes from 19 geographical regions within the country. Our study provides a refined description of the contemporary Y chromosome landscape in Finland, revealing geographical heterogeneity and novel subbranches especially within haplogroup N1a1. Overall, our findings suggest that the genetic population history of Finns may be more complex than previously suggested.

## Materials and methods

### Samples

The data for the present study was acquired from the THL Biobank (study numbers BB2019_44, THLBB2022_28) and originated from the FINRISK Project, which is a cross-sectional study of the Finnish working age population, initiated in 1972 and been carried out in 5-year cycles until 2012 [16]. Our data set consisted of 1833 men, whose sex was determined by the registry information, born between 1923 and 1979, from FINRISK surveys 1992, 1997, 2002, 2007 with whole-genome sequencing (WGS) data for the Y chromosome (Table S1). For each individual, the data included information on their Y chromosome sequence, birthplace, and age. For autosomal analyses we additionally had data available from FINRISK survey 2012. These data types are all described in detail later in the methods in their respective sections. All study participants have given a written consent.

### Whole genome sequencing data quality control

WGS was performed for a subset of the FINRISK participants (*N* = 3322 males and females) at the University of Washington using target coverage of 20x. The reads were mapped to GRCh38, and variant calling was performed. Only variant calls for the Y chromosome were acquired for this project, comprising 1833 male samples and 295,292 Y-chromosomal variants. 117,536 of these sites were non-polymorphic in the FINRISK data since variant calling was performed with additional samples not part of the FINRISK cohorts. We performed variant and sample-wise quality control for the data. We set all heterozygous calls as missing and excluded variants without PASS filter. We excluded regions with poor read mappability as described previously [17], and kept only polymorphic SNP calls with genotyping quality ≥95%. We further removed variants with mean depth <10, strand bias FS > 13, and mapping quality <50, overall yielding in 15,012 SNPs after quality control. We removed two samples that had more than 75% of missing data.

We compared the allele frequency concordance with three WGS datasets of Finns (Supplementary methods). The majority of the variants were found in these reference data sets (14,235 variants; 95%) with concordant frequencies (Fig. S1).

### Geographical location of the samples

Geographical location of the samples was determined by mapping the samples into 19 geographical regions, comprising 18 current administrative regions within Finland and one former Finnish region (Ceded Karelia) (Fig. 1). For most of the samples, we utilized the father’s birthplace as the geographical origin (*N* = 1427), since this enables to map the samples one generation back in time and limits the effects of recent population movements. The remaining samples missing information of their parental birthplaces (*N* = 400), were mapped into the regions using their own birthplace. We removed samples with missing geographical data (*N* = 4), samples having their birthplace abroad (*N* = 24), and one individual from Åland due to the low coverage of the region, overall leaving 1802 samples in the dataset (Fig. S2).

### Haplogrouping and phylogenetic analyses

To identify phylogenetically informative variants in the data, and thereby Y-chromosomal haplogroups, we built a phylogenetic tree using all variants with at least 5 carriers (i.e., MAC ≥ 5) in the data. We restricted the analyses to these variants since the focus was to describe Y-chromosomal haplogroups commonly observed in the population, and to avoid reporting rare variants for data privacy reasons. The phylogenetic relationships of these variants were resolved using SNPtotree [18] (Supplementary methods). We refer to the identified clades of the phylogenetic trees as Y-chromosomal haplogroups and use these in all following analyses.

We further used the phylogenetic tree to estimate the times to the most recent common ancestor (TMRCA) by calculating the number of derived mutations on the haplogroup clades of interest [19] (Supplementary methods).

### Haplogroup nomenclature

We refer to the haplogroups using the naming convention from the YFull database [20], i.e., referring to the haplogroups by the main one-letter haplogroup and its defining marker (Supplementary methods). To further assist in linking findings across previous studies and other databases, we provide the haplogroup alias names according to different naming conventions in the supplementary tables (Tables S2–S5).

### Haplogroup frequencies

We calculated frequencies for all haplogroups identified from the phylogenetic trees. In addition to these raw haplogroup frequencies, we also calculated scaled haplogroup frequencies that were normalized using regional population sizes from Statistics Finland [21]. This was done because we had an excess of samples from Eastern Finnish regions in our data (Fig. 1) and we aimed to provide less biased frequency estimates for the whole country. For the scaled frequencies, we first calculated the haplogroup frequencies per region and then took the weighted average of all regional frequencies. The scaled frequencies are used throughout the text when mentioning haplogroup frequency estimates in Finland.

### Geographical enrichment

We assessed the geographical distributions of all Y-chromosomal haplogroups with least within 1% in the data. The regional frequencies were calculated across each of the 19 regions. We tested the regional enrichments by a *X*^2^ test with equal frequencies across all regions as the null hypothesis. We used the whole dataset of 1802 samples in our main analyses, and further validated the results using a set of 1650 unrelated samples (Supplementary methods).

We calculated regional frequencies out of all samples (e.g., I-L258 out of all samples), and out of major haplogroup carriers (e.g., I-L258 out of I1a carriers), and further visualized both these enrichments on a regional level (online figures). To protect sample privacy and to provide more consistent frequency estimates, we used regional averaging on low coverage regions (Supplementary methods).

### Autosomal genetic background

As earlier studies have identified strong autosomal genetic structure in Finland [6], resembling that of the distribution of the main Y chromosome haplogroups [7], we wanted to assess if there is correlation between these two genetic components. To this end, we assessed autosomal principal components (PCs), computed for 1709 samples (Supplementary methods), and compared their distributions between carries of different Y-chromosomal haplogroups. To define the autosomal genetic backgrounds of the Y chromosome lineages to a finer detail, we additionally assessed pre-defined autosomal ancestry profiles from Kerminen et al. (2021) for 485 samples (Supplementary methods), where the major source of ancestry is accurately detected three generations back in time.

### Comparing the autosomal genetic background of Finns to Estonians

To further quantify the genetic relatedness of Finns in each geographic region to the closest neighboring population, Estonians, we conducted a PC analysis using 23,443 Finnish samples from the FINRISK study and 32 genotyped Estonian samples acquired from Tambets et al. (2018) (Supplementary methods). Using the same samples, we further calculated the pairwise-F_ST_ between all Finnish regions and Estonia (Supplementary methods).

## Results

### Major Y-chromosomal haplogroups in Finland

To characterize the landscape of Y-chromosomal variation in Finland, we analyzed Y-chromosomal sequencing data for 1802 geographically mapped men from the FINRISK project [16]. The samples were first assigned into a major haplogroup using YLineageTracker [22] (Table S2), and detailed sublineages and their relationships among these were identified with phylogenetic analyses with SNPtotree [18] (Tables S3–S6). With such extensive dataset, we were able to refine estimates of Y-chromosomal haplogroup frequencies in Finland by scaling them by regional population sizes (see Methods) (Table S6). We refer to these scaled frequencies throughout the results.

**Fig. 2:**
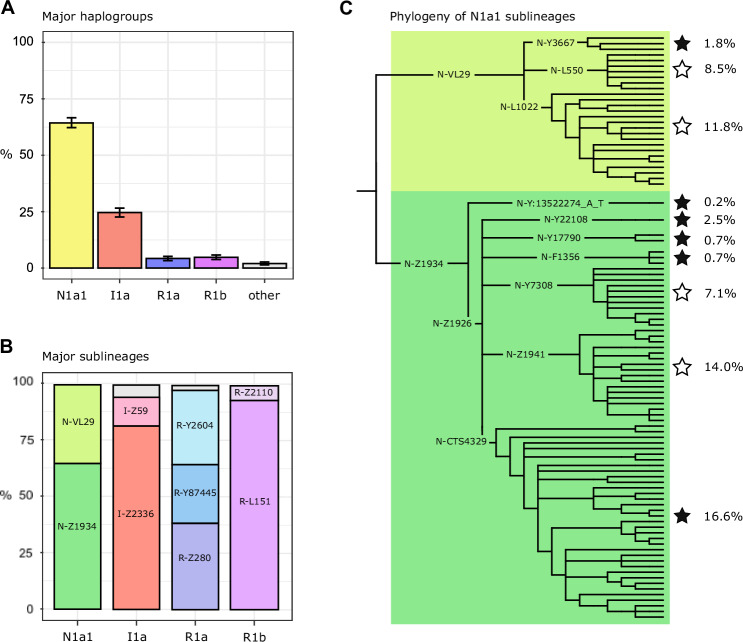
Y-chromosomal variation in Finland. **A** Scaled frequencies of the major haplogroups in Finland. Classification of “other” contains haplogroups I1f, I2, N1a2, E, J, Q, T rare among Finns. **B** The major sublineages detected among each major haplogroup presented in scaled frequencies. The gray areas represent less common lineages observed, which for I1a encompasses lineages I-Z63 and I-Y11205, and for R1a lineage R-Z2125. **C** Phylogenetic presentation of common sublineages identified within haplogroup N1a1 among Finns. Each branch corresponds to a haplogroup clade observed within at least 5 samples. The frequencies indicated next to the phylogeny correspond to scaled frequencies in our data. The white stars indicate lineages that have previously been observed by Ilumäe et al. (2016), while black stars correspond to lineages not observed within that study.

As reported previously for Finnish Y chromosomes, the majority of our samples distributed among major haplogroups N1a1 (64.3%), I1a (24.6%), R1a (4.3%), and R1b (4.8%) (Fig. 2A; Table S6), with similar frequency estimates to previous studies [7, 9]. The remaining 2% of men not belonging to these major haplogroups carried haplogroups I1f, I2, N1a2, E, J, Q, T (Table S2).

### Substructure beyond the major haplogroups

Beyond the major haplogroups, our data enabled the identification of further sublineages (Fig. 2B, C; Tables S3–S6). Within haplogroup N1a1, we observed a major division into into two lineages, N-VL29 (22.2%) and N-Z1934 (42.2%) (Fig. 2B, C; Table S3). Beyond this, N-VL29 divided into three sublineages (Fig. 2C; Table S3), with their TMRCA estimates ranging from 2500 to 3430 ya (CI 2130–3950) (Table S7). Within haplogroup N-Z1934, most of its carriers belonged to N-Z1926, which then divided into six sublineages (Fig. 2C; Table S3), with their TMRCA estimates ranging from 1720 to 2580 ya (CI 1470–2970) (Table S7).

Within haplogroup I1a, we observed a split into haplogroups I-Z2336 (20.1%), I-Z59 (3.1%), and I-Z63 (0.4%) (Fig. 2B; Table S4), as previously described among Finns [9]. The most common sublineage descending from I-Z2336 was I-L258 (16.7%), which further divided into 13 further sublineages among Finns (Table S4).

Within haplogroup R1a we found that the samples divided into three sublineages: R-Z280 (1.7%), R-Y87445 (0.9%), and R-Y2604 (1.5%). R1b divided into two sublineages: R-L151 (4.6%) and R-Z2110 (0.3%) (Fig. 2B; Table S5) in our data.

### Identifying novel haplogroups

Besides observing these previously annotated haplogroups among Finns, our phylogenetic trees were further able to resolve and identify a number of novel haplogroups. Of the 363 haplogroup clades detected, 104 (28.7%) did not have any previous annotation in ISOGG (v15.73) [23] or YFull (v10.01) [20] databases (Table S6). The majority of the novel subbranches were observed within haplogroup N1a1 (81%) (Table S3; Table S6). For instance, we found that the relatively common haplogroup N-CTS7189 (4.4%) divided into six sublineages, of which four were completely novel, and within two we resolved further phylogenetic relationships of novel and known sublineages (Table S3).

### Major haplogroups N1a1, I1a, and R1a show east-west differences

Next, we selected haplogroups having a frequency of at least 1% in the data (*N* = 103), to test for their geographical enrichment (Tables S8 and S9; online figures). We assessed the geographical distributions by the regional frequencies corresponding (1) to each haplogroup’s proportion of all samples (Table S8), and (2) to each haplogroup’s proportion of its major haplogroup (N1a1, I1a, R1a or R1b) (Table S9).

**Fig. 3:**
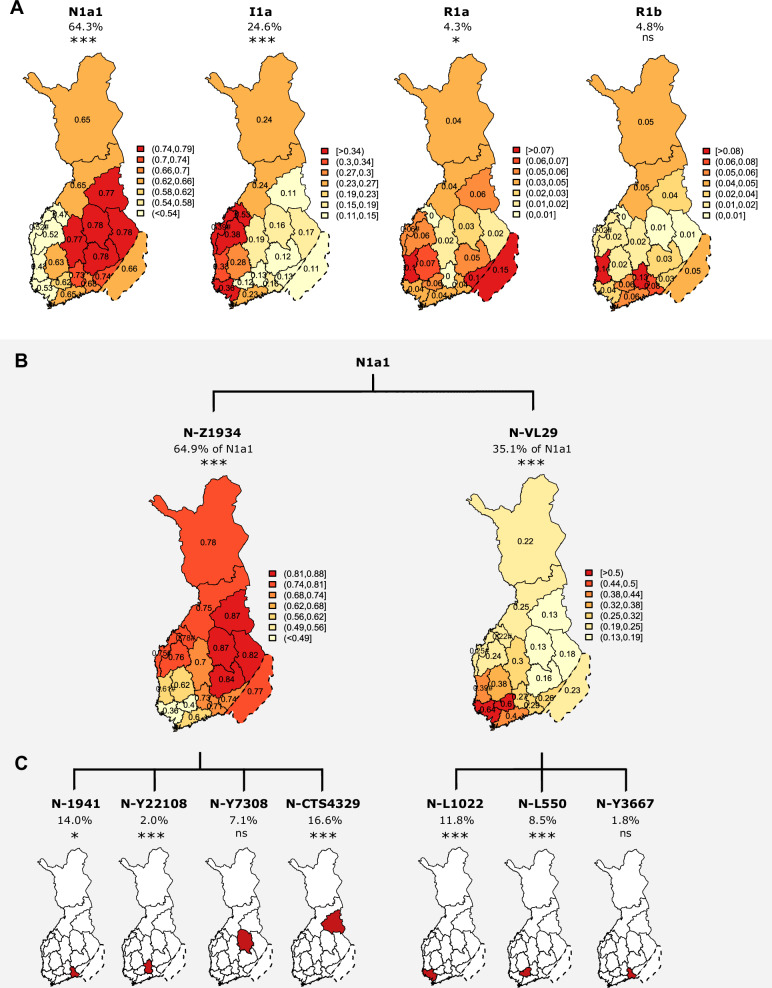
Geographical distribution of Y-chromosomal haplogroups in Finland. **A** Major haplogroups N1a1, I1, R1a, R1b, with the haplogroup frequencies calculated out of all samples. **B** The division of N1a1 into two major sublineages. The haplogroup frequencies are calculated out of the major haplogroup N1a1. **C** Most common sublineages within N-Z1934 and N-VL29, with the maps highlighting only the area with highest observed frequency. Within panels (**A**) and (**B**), the coloring indicates haplogroup frequency and is scaled for each map separately with the average frequency as the midpoint. Stars within the figure titles correspond to *p*-value for *X*^2^ test for geographical enrichment (ns=not significant, *=nominally significant, ***=significant after multiple testing correction). A hashtag (#) within a map indicates the frequency is inferred by combining samples from geographically closest regions due to low coverage of samples in the region (in panel A for the OST, and within panel B for OST, CO and SAT).

As previously reported [7, 9], we observed strong geographical enrichment for haplogroups N1a1 and I1a (Fig. 3A; Table S8). Haplogroup N1a1 reached its highest frequency of 78% in North Karelia, North Savonia, and Southern Savonia (*p* = 3.1 × 10^–7^), (Fig. 3A; Table S8). In contrast, haplogroup I1a displayed an enrichment along the western coast of Finland, reaching its peak frequency of 53% in Central Ostrobothnia (*p* = 5.2 × 10^–7^) (Fig. 3A; Table S8). In addition to these previously established findings, we discovered nominally significant heterogeneity in the geographical distribution of haplogroup R1a (*p* = 1.2 × 10^–3^), enriched in Ceded Karelia (15%) and Satakunta (10%) (Fig. 3A). Although haplogroup R1b reached its highest frequencies in Satakunta (14%) and Päijät-Häme (13%) (Fig. 3A), the result was not statistically significant (Table S8).

### Substantial regional heterogeneity beyond the major haplogroups

Assessing the distribution of sublineages within the major haplogroups (N1a1, I1a, R1a, and R1b), we observed 41 sublineages showing regional heterogeneity (*p* < 0.05, *X*^2^ test for equal proportions, df = 18), with 18 of these remaining significant after multiple testing correction (*p* < 0.05/96) (Table S9). The majority of these enrichments (*N* = 33) were related to haplogroup N1a1 sublineages.

Out of the two N1a1 sublineages (Fig. 3B), haplogroup N-Z1934 (42.2%) displayed enrichment to the east (*p* = 1.7 × 10^–17^) (Fig. 3B, Table S9), aligning with the overall geographical enrichment pattern of the major haplogroup N1a1 (Fig. 3A). Two of the N-Z1934 sublineages displayed significant enrichment, with CTS4329 (16.6%), enriched in Kainuu (55% of N1a1, *p* = 7.5 × 10^–6^), and N-Y22108 (2.0%) in Päijät-Häme (18% of N1a1, *p* = 4.1 × 10^–7^). In addition, N-Z1941 (14%) displayed nominal enrichment in Kymenlaakso (47% of N1a1, *p* = 4.8 × 10^–2^) (Fig. 3B).

In contrast, haplogroup N-VL29 (22.2%) (Fig. 3B), displayed enrichment to the opposite side of the country, reaching its highest frequency of 64% of N1a1 in Southwest Finland (*p* = 9.8 × 10^–5^) (Fig. 3B; Table S9). Two of its sublineages displayed a fairly similar enrichment pattern, with N-L1022 (11.8%) enriched in Southwest Finland (44% of N1a1, *p* = 5.5 × 10^–6^) and N-L550 (8.5%) enriched in Tavastia proper (30% of N1a1, *p* = 5.5 × 10^–13^) (Fig. 3B).

Within haplogroup I1a sublineages, we observed one significant enrichment for I-Y17288 (1.6%), reaching its highest frequency in the west (33% of I1a in South Ostrobothnia, *p* = 1.3 × 10^–4^). For six further I1a lineages we observed a nominal enrichment, of which four were enriched to western regions similarly to the major haplogroup, while two were enriched to the east (Table S9).

For sublineages among haplogroups R1a and R1b, we could not find geographical enrichments. Although haplogroup R-L151 (4.6%) displayed nominal evidence for heterogeneity (*p* = 0.013), it did not suggest a clear centralized area of enrichment (Table S9). Within haplogroup R1a, which overall displayed a dual enrichment in the east and the west (Fig. 2A), we observed that lineages R-Y2604 and R-Z280 displayed higher frequencies in the east, while for instance R-Y35 was more common in the west, although these enrichments were not statistically significant (Table S9; online figures).

### Autosomal genetic structure correlates with haplogroups N1a1 and I1a in Finland

**Fig. 4:**
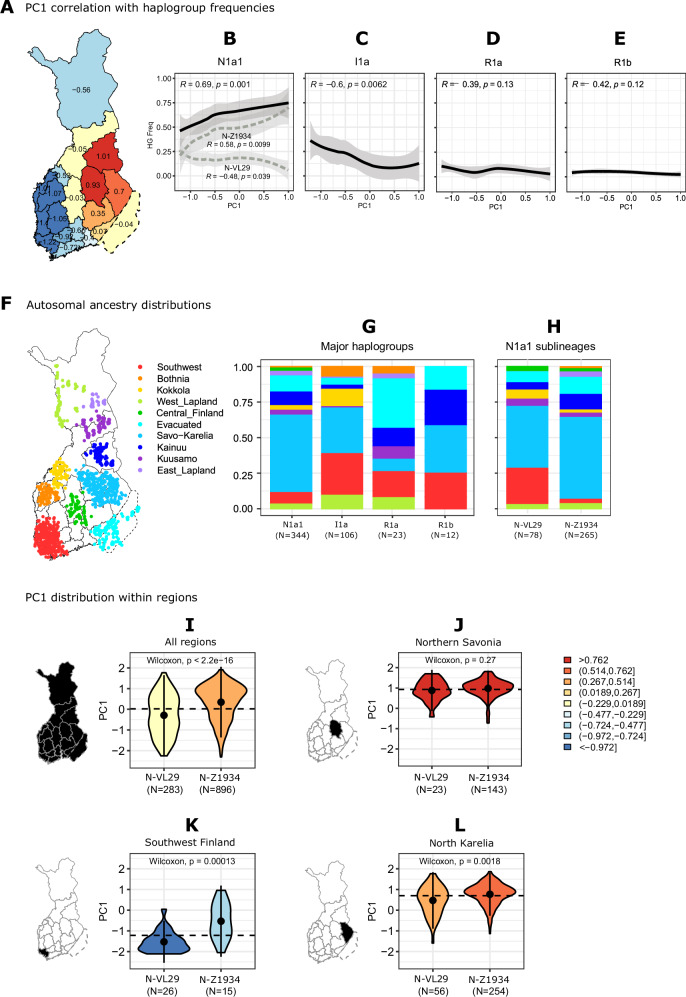
Connection between the autosomal genome and Y chromosomal haplogroups. **A**–**E** The correlation between autosomal PC1 and Y-chromosomal haplogroup frequencies regionally for the major haplogroups. The lines are visualized by LOESS. **F**–**H** Autosomal ancestry distributions by 10 FineSTRUCTURE reference populations from Kerminen et al. (2021), with the coloring ordered by the populations’ approximate FST distances. Each individual was assigned to one population if sharing at least 50% of their genome with the reference population. **I**–**L** Regional distribution of autosomal PC1 regionally compared between haplogroups N-VL29 and N-Z1934. The coloring in panels (**I**)–(**L**) corresponds to autosomal PC1 scores.

The autosomal genetic structure and Y-chromosomal haplogroups can both show strong correlations to local geography, as has been evidenced for instance in Finland [6, 7] and the UK [24, 25]. Since in Finland the autosomal genetic population structure resembles the distribution of the major Y chromosome haplogroups (Fig. 3A; Fig. 4A), we aimed to determine to which degree these two genetic components display a connection. To examine this link, potential in providing further demographic insights of the population, we compared the autosomal genetic background of Y-chromosomal haplogroup carriers (1) by autosomal PCs 1-20 (Fig. 4A), and (2) by autosomal ancestry profiles from Kerminen et al. [26] (Fig. 4F).

At the major haplogroup level, we observed a significant correlation between PC1 and haplogroups N1a1 (*R* = 0.69, *p* = 0.001) and I1a (*R* = –0.60, *p* = 0.006) (Fig. 4B, C). In contrast, haplogroups R1a and R1b did not show significant correlation with the PC1 (Fig. 4D, E), consistent with their more dispersed enrichment patterns. When assessing the PC1 correlation within the N1a1 sublineages, we observed the correlation pattern of the southwestern N-VL29 was opposite to that of the main haplogroup N1a1 (*R* = –0.47, *p* = 0.043) (Fig. 4B). This suggests the correlation between the autosomal PCs and Y-chromosomal haplogroups captured on the major haplogroup level is not necessarily representative of the relationship within the further sublineages.

### N1a1 sublineage carriers show distinct autosomal genetic ancestry

As a complementary approach, we employed autosomal ancestry profiles from 10 reference populations acquired from Kerminen et al. [26] (Fig. 4F, G). As expected, most of haplogroup N1a1 carriers belonged to the eastern Savo-Karelia ancestry, while carriers of haplogroup I1a displayed higher proportions of western ancestries (Figs. 4G and S3). Within haplogroup R1a we observed the highest proportion of Evacuated ancestry (i.e., individuals evacuated from southeast Finland during the second world war) [26] (Figs. 4G; and S3). Haplogroup R1b displayed visually higher proportions of Kainuu ancestry (Fig. 4G), although the proportion was not significantly higher than for R1a or N1a1 (Fig. S3), likely impacted by the small sample size for R1b (*N* = 12) in the analyses.

When comparing the autosomal genetic backgrounds of the two N1a1 sublineages (Fig. 4H), we observed that lineage N-Z1934 was enriched in Savo-Karelia ancestry (Fig. 4G), whereas the southwestern lineage N-VL29 displayed a higher proportion of Southwest ancestry (Fig. 4H). As an exception, within the sublineages of N-VL29, carriers of N-Y3667 displayed a lower proportion of Southwest ancestry than N-VL29 in general (Fig. S4), and higher proportions of Savo-Karelia and Evacuated ancestries, in a similar manner to N-Z1941 (Fig. S4).

### Regional differences in PC1 indicate recent population movements

To further investigate a possible southwestern origin for haplogroup N-VL29, we compared the distribution of autosomal PC1 scores between carriers of the southwestern N-VL29 and eastern N-Z1934 within individual regions (Fig. 4I–L). In Southwest Finland, we observed significant differences between the PCs for the carriers of haplogroups N-VL29 and N-Z1934 (Wilcoxon *p* = 1.3 × 10^–4^) (Fig. 4K), suggesting distinct demographics for these two haplogroups within this region. Carriers of the southwestern lineage N-VL29 were enriched towards lower PC1 values (typical for samples of southwestern origin), whereas carriers of the northeastern lineage N-Z1934 were enriched towards higher PC1 values (typical for northeastern regions). Similar differences between these haplogroups were further observed in North Karelia (Wilcoxon *p* = 1.8 × 10^–3^) (Fig. 4L). Collectively these findings highlight the differences in the autosomal genetic characteristics of men bearing Y-chromosomal haplogroup N1a1, supporting the idea of a possible major southwestern introduction for N-VL29 and major eastern introduction for N-Z1934 into the country.

### Finns from the Southwest are genetically closer to Estonians than the rest of the population

Given the relatively high frequency of the N-VL29 in the Baltic countries, especially in Estonia [10], we looked for evidence of a possible Estonian influence of haplogroup N-VL29 to Finland. To this end, we assessed the autosomal genetic relatedness between 23,443 FINRISK samples and 32 Estonian samples [27] by a joint PCA, and further quantified the relationship by pairwise-F_ST_.

In the PCA space we observed that Estonian samples positioned closest to samples from southwestern regions of Finland, such as Southwest Finland and Satakunta (Fig. S5). Further assessing the genetic distances by *F*_ST_, we observed on average the genetic distance between Estonians and Finns was greater than any of the observed differences between Finns (Table S10). However, the Finnish region closest to Estonians was Southwest Finland (*F*_ST_ = 279.2 × 10^–5^, SE = 19.9 × 10^–5^) where the *F*_ST_ assessment indicated similar or even closer genetic similarity between the two populations than between the samples from the western and eastern parts of Finland (Table S10). As such, these results lend further support to the idea of a possible southwestern introduction for haplogroup N-VL29 into Finland.

## Discussion

In this study, we set out to study the Y-chromosomal landscape in Finland by using Y-chromosome sequencing data from 1802 Finnish men born between 1923 and 1979. Our data reflects the Y-chromosomal landscape in Finland from the beginning of the 20th century to approximately the 1950’s, before the start of the large-scale internal movements and urbanization within Finland. The resolution of our data enabled subdivision of previously described major haplogroups (N1a1, I1a, R1a, R1b) in Finland into numerous sublineages common in the population, and the identification of lineages not previously reported in comprehensive Y chromosome databases [20, 23]. Collectively, our findings indicate a more complex population history for Finns than shown in previous genetic studies.

The majority of Finnish men carry haplogroup N1a1 (64% our estimate), which has been shown to be frequent especially in eastern Finland [7, 9]. Our data highlights the specific contribution of the haplogroup N-Z1934 (42.2%) driving this enrichment pattern. N-Z1934 descends from haplogroup N-Z1936 that is found in Hungarian samples from the 10th century, and hence associated as a plausible connection among members of the Finno-Ugric language family [28]. Globally, N-Z1934 is known to show high frequency among Finns, reaching notable frequencies also in the neighboring populations towards the east, e.g., among Vepsas, Karelians, Saamis, and North Russians [29].

Given the eastern enrichment of N-Z1934 in Finland, its likely source to the country is through migrations from Siberia and the Volga-Ural region [12] that first reached the Northeastern Europe around 3500 years ago [30]. Theoretically, the distinct geographical enrichment patterns for the sublineages of N-Z1934 can suggest multiple introduction events, internal migration, or combination of both. While the lineages N-CTS4329 (16.6%) and N-Y7308 (7.1%) display their peak frequencies in the eastern regions of Kainuu and Northern Savonia, lineages N-Z1941 (14%) and N-Y22108 (2%) are most frequently observed in the southeast. Observing sublineages present among Russians for N-Y7308, and among Estonians and Saami for N-Z1941 [10], supports potentially distinct introduction events of these lineages to Finland. Further, the wide dispersal of the North Savonian lineage N-Y7308 throughout the country may reflect the Savonian population expansion during the late settlement process of Finland [31]. Further, this expansion serves as a likely explanation for the specific enrichment of lineage N-CTS4329 (16%) enriched in Kainuu [32].

In addition to the eastern enriched N1a1 lineages, approximately one-third of N1a1 constitutes of haplogroup N-VL29 (22%). Globally, recognized as the Baltic branch of N1a1, this haplogroup reaches its highest frequencies among Estonians (28%) and is further present among Latvians, Lithuanians, Finns, Saami, Karelians, Belarusians, Ukrainians, Russians [10]. In Finland, this haplogroup displays strong geographical enrichment to the southwestern coast of Finland, with the haplogroup carriers on average exhibiting a higher proportion of southwestern autosomal genetic ancestry compared to the N-Z1934 sublineages. This heterogeneity within haplogroup N1a1 suggests distinct arrival routes for N1a1 into Finland, instead of a single founder population from the east as previously proposed [7, 15].

Although N-VL29 has its origin in the Volga-Uralic region in a similar manner to N-Z1934 [10], its major source to Finland is suggestively from Estonia, where this haplogroup is most frequent [10]. This hypothesis is further supported by our observation that Estonians display the closest autosomal genetic connection to Southwestern Finns. Overall, Finns and Estonians share close historical, linguistic, and genetic connections with each other. Notably, the Southwestern Finnish dialect spoken within the area stands out for its similar features to the Estonian language in comparison to other Finnish dialects [33]. This potential genetic influence from Estonia could originate from a relatively recent event such as the late migration from Estonia to Finland around 1300 to 1100 years ago [34], although we cannot determine the arrival time of the haplogroup into the country by utilizing only contemporary DNA. However, since N-VL29 has also been observed among Swedes already during the Viking ages [35, 36], although having only an overall frequency of 4.4% nowadays in Sweden [37], we cannot exclude a more complex pattern of migration affecting the enrichment of the haplogroup in Southwest Finland based on the data of this study.

Beyond haplogroup N1a1, 25% of Finnish men carry haplogroup I1a. As reported previously, haplogroup I1a reaches its highest frequencies along the western coast of Finland. This pattern is in concordance with the suggested Scandinavian influence of this haplogroup into the country [7], coinciding geographically with the early settlement region to the coastline of Finland [4]. While we find a predominantly western enrichment for the majority of the haplogroup I1a lineages, we distinguished an eastern enrichment for haplogroup I-Y10990 and its sublineages, carried by 2.3% of Finnish men. Although this haplogroup has been observed at a zero frequency among Swedes [35], it descends from I-L258, which is a known as a Finnish enriched I1a lineage [9], therefore most likely reflecting a dispersal from western Finland to the eastern regions of the country.

Most of the remaining Finnish men carry haplogroups R1a-L62 (4.3%) and R1b-CTS2134 (4.8%), suggested to reflect Eastern and Western European ancestry contributions [15, 38, 39]. We find that haplogroup R1a is geographically enriched in the east but also reaches high frequencies locally in the west. Within R1a we observe sublineages previously observed in Russia, Baltics, and Scandinavia [40], supporting the hypothesis of haplogroup R1a influence from both directions to the country as previously suggested [15]. For haplogroup R1b we did not find any significant enrichment, implying a relatively equal spread across the country, although the lack of the any observed enrichments could partly be influenced by the relatively small sample size for this haplogroup within our data.

In summary, we provide a comprehensive exploration of Y-chromosomal variation in Finland, unraveling finer-scale variation in the population, in particular related to haplogroup N1a1. We suggest that haplogroup N1a1 most likely arrived to the country via two distinct routes: from the eastern paths through the mainland, and the southwestern direction via the Baltic Sea. Overall, our results highlight that studying the paternally inherited Y chromosome using WGS data mapped to precise geographical origins has potential to capture additional population historical events compared to autosomal genetic data alone.

## Supplementary information

Supplementary Methods and Materials

Supplementary Table S1

Supplementary Table S2

Supplementary Table S3

Supplementary Table S4

Supplementary Table S5

Supplementary Table S6

Supplementary Table S7

Supplementary Table S8

Supplementary Table S9

Supplementary Table S10

## Data Availability

The data used in this study is available through the Finnish Institute for Health and Welfare (THL) Biobank (http://www.thl.fi/biobank). Online figures: results for Y-chromosomal regional enrichment maps for all common haplogroups are available at 10.5281/zenodo.13903322 and 10.5281/zenodo.13904061. Supplementary material includes Figures S1–S5 and Tables S1–S10. This is an open-access article distributed under the terms of the Creative Commons Attribution 4.0 International License (https://creativecommons.org/licenses/by/4.0/), which permits unrestricted use, distribution, and reproduction in any medium, provided the original work is properly cited.
