# Supplementary material for: Y chromosome sequencing data suggest dual paths of haplogroup N1a1 into Finland
Source: Eur J Hum Genet. 2024 Oct 28;33(1):89–97. doi: 10.1038/s41431-024-01707-7 (PMC11711460; doi:10.1038/s41431-024-01707-7)
Supplement: Supplementary file 1 — Supplementary Methods and Materials [file 41431_2024_1707_MOESM1_ESM.pdf]

SUPPLEMENTARY METHODS

Reference WGS datasets for allele frequency comparison

We assessed the allele frequencies in three WGS datasets of Finns: Sequencing Initiative Suomi (SISu) v4 (N=7,019)[1], the Genome Aggregation Database (gnomAD) v3.1.2 (N=4,029)[2] and to a combined resource from the 1000 Genomes Project and Human Genome Diversity Project data (1kGP+HGDP) (N=38)[3]. The sequencing data from 1kGP+HGDP was filtered with the same criteria as our FINRISK data, leaving 6,229 variants and 38 Finnish samples in the data. For SISu and gnomAD, which also contain samples from the FINRISK study, we only acquired variant level summary data. SISu variants were filtered with call rate ≥95%, heterozygous call rate ≤0.01, removing indels and non-polymorphic variants, yielding 68,624 variants (called for 7,019 Finnish samples). gnomAD variants were filtered to exclude non-polymorphic variants, yielding 44,553 variants (called for 4,029 Finnish samples). Most of the variants identified in our data were found in these reference data sets (14,235 variants; 95%) with good allele frequency concordance (Figure S1).

Overall, 92% of the Y-chromosomal variants passing quality control were rare (MAF ≤ 0.01), with 64% of all variants being singletons. 4,778 variants (32%) were annotated as haplogroup-defining either in ISOGG (v15.73)[4] or YFull (v10.01)[5].

Comparison of YLineageTracker haplogroups to SNPtotree

We assigned samples first to haplogroups by YLineageTracker[6]. However, we noticed that many of the assigned haplogroups (21.5%; 64/297), had more than

five carriers, overall implying further substructure among these haplogroups not detected by YLineageTracker. To this end, we utilized SNPtotree[7], which allowed to exploit variations beyond the ISOGG database[4], when resolving the samples' phylogenetic relationships and consequently determining their haplogroups.

We ran SNPtotree[7] with options "-contradictory_variants -ambiguous_variants -metadata_individuals". We built phylogenies separately within carriers of haplogroups N, I, and R to reduce the computational load of the analysis.

We annotated the haplogroup clades within the phylogeny using variant annotations from Y chromosome databases YFull (v10.01)[5] and ISOGG (v15.73)[6], and from previous Y chromosome studies of Karmin et al. (2015) and Ilumäe et al. (2016). We assigned names primarily by the YFull annotation, but for clades that had a different annotation available, we assigned a name similarly using the major haplogroup and its defining marker. For clades that did not have any previous annotation available, we refer to these with the main haplogroup, variant position (GRCh38), and reference and alternative alleles.

To compare the haplogroups determined by these two methods, we assessed where the samples with a certain YLineageTracker haplogroup located among the phylogenetic trees. We identified that 21% of the assigned YLT haplogroups (63/296) divided into 2 or more common sublineages among the phylogenies, overall implying a better haplogroup resolution for these lineages using SNPtotree. For instance, YLineageTracker assigned haplogroup N-CTS7189 (N1a1a1a1a2a1a1a1a2c~) for 126 samples in our dataset, while the phylogenetic analyses were able to divide carriers of N-CTS7189 into six further sublineages.

Beyond resolving haplogroups to further sublineages, we also observed haplogroup misclassification by YLineageTracker due to contradicting variants in ISOGG. For instance, haplogroup N-A12258 (N1a1a1a1a1a2a3a~) was assigned to 43 samples by YLineageTracker, although the phylogenetic analyses revealed that only 6 samples belonged to this lineage, and the remaining 37 samples belonged to a parallel branch of N-Y10756 (N1a1a1a1a1a2a1a1) (Table S2). Majority of these unresolved lineages in YLineageTracker were observed for haplogroup N1a1 (N=47), implying the current ISOGG database lacks a significant number of haplogroup-informative variants for N1a1.

Haplogroup regional averaging

We calculated regional haplogroup frequencies within each region out of all samples and out of major haplogroup carriers. To protect sample privacy and to provide more consistent haplogroup frequency estimates on low coverage regions, we used regional averaging to provide the haplogroup frequency estimate. This regional averaging was performed by adding all samples from the geographically closest region(s) to the target region until reaching a certain threshold of samples (≥ 15 samples per region, or ≥ 10 major haplogroup carriers per region) and calculating the frequency estimate using this combined set of samples. The visualizations were performed in R utilizing maps from geoBoundaries package[8]. We note that the rarest haplogroups (e.g., those with close to 1% frequency) may be inaccurately visualized on a regional level due to the regional averaging.

Confidence intervals for haplogroup frequencies

We calculated 95% confidence intervals for the raw and scaled frequency estimates by the following equation, where p is the haplogroup proportion and n is the total sample size:

$$95\%\ CI = p \pm 1.96\sqrt{\frac{p(1-p)}{n}}$$

Autosomal data quality control

To assess the autosomal genetic background of Finns, we assessed autosomal imputed genotyping data available altogether for 30,867 FINRISK samples (including males and females) genotyped with multiple arrays (Table S1). Imputation was carried out by using the population-specific SISu v3 imputation reference panel with Beagle 4.1 (version 08Jun17.d8b)[9] as described in the following protocol: dx.doi.org/10.17504/protocols.io.nmndc5e. Before quality control we had 16,962,023 autosomal variants available. We performed variant and sample-wise quality control for each chromosome separately in PLINK 2.0[10,11] removing variants with INFO < 0.95, genotyping quality < 0.99, HW p-value < 1e-6, sites with > 1% missing data and multiallelic sites. To obtain a set of independent variants, LD-pruning was performed with 1,000 kb windows, step size 1 and with r2 threshold of 0.2. After these variant filtering steps, sample-wise quality control was performed by removing individuals if they were born abroad, had missing birth region information, or had excess heterozygosity (F-statistics deviating more than 4SD units from the mean). After quality control the dataset

consisted of 23,865 samples and 119,455 variants. To link the Y-chromosomal variation with autosomal genetic variation, we assessed selected samples with Y-chromosomal sequencing data available, resulting in 1,710 male samples and 119,455 autosomal variants.

Sample relatedness

Sample relatedness was inferred in PLINK 2.0[10,11] for the 1,709 samples with autosomal genetic data available. In total 32 sample pairs were classified as closely related (3 pairs as 1st degree, and 29 pairs as 2nd degree), whereas 1,677 samples were classified as unrelated (KING kinship coefficient < 0.0442). Since 122 samples were lacking autosomal data, we estimated the number of expected relationships that may be present in the whole data. Among our autosomal samples, the rate of 1st degree related pairs was 3/(1709*1708/2) and rate of 2nd degree related pairs was 29/(1709*1708/2), thus we expect that in our whole data of 1,802 we should detect 3.3 of 1st degree and 32.2 of 2nd degree related sample pairs, numbers unlikely to bias the haplogroup frequencies estimated with the whole data. Therefore, we used the whole dataset comprising the 1,802 samples in our main Y-chromosomal analyses, and further utilized the confirmed set of unrelated samples for validating the results. From the unrelated data set we included 1,650 samples in our validation analyses, since these were part of our quality control passing Y-chromosomal dataset having their paternal birthplaces in Finland.

PCA

We performed autosomal principal component analysis (PCA) for the 1,709 samples. We first performed PCA in PLINK 2.0[10,11] for a subset of unrelated samples with autosomal data (N = 1,604) and used the output further in projecting PC scores for all 1,709 samples with autosomal data available. We then used the autosomal PCs to compare their distributions between the carries of different Y-chromosomal haplogroups and performed correlation analysis between the PC scores and Y-chromosomal haplogroup frequencies. The PCs were mapped to geographical regions using the samples' own birthplaces (Figure S2), since using only the father's birthplace is an inaccurate measure for autosomal genetic origin.

Autosomal ancestry profiles

We assessed pre-defined autosomal ancestry profiles from Kerminen et al. (2021), which were based on 10 genetically and geographically mapped Finnish reference populations, available for 758 samples in our data. We assessed the major source of ancestry for each sample by the criteria of sharing at least 50% of their genome with one reference population, resulting in 485 samples with one major source of ancestry. We used these autosomal ancestry profiles to compare their distributions between different Y-chromosomal haplogroup carriers.

TMRCA estimation

We estimated time to most recent common ancestor (TMRCA) by calculating the average number of newly acquired mutations within the subclades of a given

haplogroup clade[12]. We used two methods to derive the mutation rate, and thus the TMRCA estimates from the calculated number of mutations. We utilized a calibration point for haplogroup N-VL29 TMRCA at 4480 (3816 – 5156) ya[13] which yielded with our data a rate of 95,12 (CI: 81,02 – 109,47) years per mutation. Additionally, we derived another Y chromosome mutation rate based on the published rate of $1.0e^{-9}$ mutations per position per year (CI: $0.92e^{-9}$ – $1.09e^{-9}$)[14], generation time of 30 years, and Y chromosome length of 10,45 Mb that is uniquely mappable[15], yielding in a mutation rate of 95,69 (CI: 87,68 – 104,02) years per mutation.

We are aware that these TMRCA calculation methods rely on many assumptions. Here, we assume a constant mutation rate for the Y chromosome without incorporating an evolutionary model, which could inaccurately represent the distance to the common ancestor. Furthermore, we assume a certain sequence coverage for the Y chromosome, although having only a VCF file available (with only polymorphic positions called), thus we cannot estimate the true sequence coverage among our data. Additionally, we rely on the accuracy of the used calibration point for haplogroup N1a1, which is not dated based on fossil calibration and thus adds uncertainty to the TMRCA estimates, although we acquire with both methods fairly similar mutation rates. Nevertheless, the TMRCA estimates of the N1a1 subclades were comparable to those reported by Ilumäe et al. (2016)[13].

PCA and F_{ST} for a joint dataset of Finns and Estonians

To quantify the genetic distance between Estonians and Finns, we performed a joint PCA for all 23,443 FINRISK samples with autosomal genetic data available and 32 Estonian samples acquired from Tambets et al. (2018) that were genotyped on the 610K array[16]. We joined these two datasets together resulting in 9,932 variants found in both datasets. We performed quality control for the combined dataset, by excluding samples with excess heterozygosity (F-statistics deviating more than 4SD units from the mean) and closely related samples (KING kinship coefficient > 0.0442), resulting in 23,475 samples after quality control (23,443 Finns and 32 Estonians). We performed autosomal PCA in PLINK 2.0[10,11] for the 23,475 samples and 9,932 variants. For visualization purposes, we excluded 26 Finnish samples having their birthplace outside the 19 regions used in our study. We visualize the PCA altogether for 23,449 samples (Figure S5).

Using the same dataset of 23,449 samples, we calculated pairwise-FST using SmartPCA of EIGENSOFT (version 7.2.1) (fstonly: YES, fsthiprecision: YES) between each of the 19 Finnish regions and Estonia (Table S10).

REFERENCES

1. Sequencing Initiative Suomi project (SISu). http://sisuproject.fi Accessed 2 May 2024

2. Collins RL, Brand H, Karczewski KJ, Zhao X, Alföldi J, Francioli LC, et al. A structural variation reference for medical and population genetics. Nature. 2020 May 28;581(7809):444–51.

3. Koenig Z, Yohannes MT, Nkambule LL, Goodrich JK, Kim HA, Zhao X, et al. A harmonized public resource of deeply sequenced diverse human genomes. bioRxiv. 2023 Aug 10;2023.01.23.525248.

4. International Society of Genetic Genealogy (ISOGG) v15.73. https://isogg.org Accessed 11 Jan 2022.

5. YFull v10.01. https://www.yfull.com/tree/ Accessed May 1 2024.

6. Chen H, Lu Y, Lu D, Xu S. Y-LineageTracker: a high-throughput analysis framework for Y-chromosomal next-generation sequencing data. BMC Bioinformatics. 2021 Dec;22(1):114.

7. Köksal Z, Børsting C, Gusmão L, Pereira V. SNPtotree—Resolving the Phylogeny of SNPs on Non-Recombining DNA. Genes. 2023 Sep 22;14(10):1837.

8. Runfola D, Anderson A, Baier H, Crittenden M, Dowker E, Fuhrig S, et al. geoBoundaries: A global database of political administrative boundaries. PLoS One. 2020;15(4):e0231866.

9. Browning BL, Browning SR. Genotype Imputation with Millions of Reference Samples. The American Journal of Human Genetics. 2016 Jan;98(1):116–26.

10. Purcell Shaun, Chang Christopher. PLINK 2.0. www.cog-genomics.org/plink/2.0/

11. Chang CC, Chow CC, Tellier LC, Vattikuti S, Purcell SM, Lee JJ. Second-generation PLINK: rising to the challenge of larger and richer datasets. GigaSci. 2015 Dec;4(1):7.

12. Forster P, Harding R, Torroni A, Bandelt HJ. Origin and evolution of Native American mtDNA variation: a reappraisal. Am J Hum Genet. 1996 Oct;59(4):935–45.

13. Ilumäe AM, Reidla M, Chukhryaeva M, Järve M, Post H, Karmin M, et al. Human Y Chromosome Haplogroup N: A Non-trivial Time-Resolved Phylogeography that Cuts across Language Families. The American Journal of Human Genetics. 2016 Jul;99(1):163–73.

14. Helgason A, Einarsson AW, Guðmundsdóttir VB, Sigurðsson Á, Gunnarsdóttir ED, Jagadeesan A, et al. The Y-chromosome point mutation rate in humans. Nat Genet. 2015 May;47(5):453–7.

15. Poznik GD, Henn BM, Yee MC, Sliwerska E, Euskirchen GM, Lin AA, et al. Sequencing Y Chromosomes Resolves Discrepancy in Time to Common Ancestor of Males Versus Females. Science. 2013 Aug 2;341(6145):562–5.

16. Tambets K, Yunusbayev B, Hudjashov G, Ilumäe AM, Rootsi S, Honkola T, et al. Genes reveal traces of common recent demographic history for most of the Uralic-speaking populations. Genome Biol. 2018 Dec;19(1):139.

SUPPLEMENTARY FIGURES AND TABLES

Figure S1: Allele frequency concordance with three Finnish WGS datasets: SISu v4, 1000 Genomes Project and Human Genome Diversity Project data Finns (TG), and gnomAD v3.1.2 Finns. We accepted the allele frequencies to differ up to 15 percentages between FINRISK and the reference data sets, since the variant frequencies are known to vary extensively based on the geographical location.

Figure S2: A) Sample distribution for Y-chromosomal analyses (N=1,802), and B) autosomal analyses (N=1,709). C) Concordance between father's and own birth region indicated as a proportion of fathers having their son born in the same region. D) Sample distribution by birth year.

Figure S3: Autosomal ancestry proportions for major haplogroups N1a1, I1a, R1a, R1b. Significance testing is based on two-sample proportion test.

Figure S4: Autosomal ancestry proportions for major N1a1 sublineages. Significance testing is based on two-sample proportion test. The autosomal ancestry estimation for haplogroup N-Y22108 may be inaccurate due to the low sample size in this analysis (N=2).

Figure S5: Genetic relatedness between Finns and Estonians. A) Geographical distribution of 23,434 FINRISK samples used for joint PCA analysis together with 32 Estonians from Tambets et al. (2018) B) PC1 vs. PC2 for the combined dataset of Finns and Estonians. The PCs are standardized (mean 0, variance 1). Gray dots represent individual samples and diamonds correspond to the mean PC score from each geographical location. C) PC1 vs. PC2 colored by each sample's geographical location. Estonian samples are visualized with black diamonds.

Online Figures

https://doi.org/10.5281/zenodo.13903322

https://doi.org/10.5281/zenodo.13904061

Geographical enrichment maps for all haplogroups with at least 1% frequency in the data. A star (*) within the map and the gray color within the forest plot indicates the frequency is inferred by combining samples from geographically closest regions due to low coverage of samples in the region. Files ending with "_all.png" correspond to frequencies out of all samples, while files with ending "_major.png" correspond to frequencies out of major haplogroup (N1a1, I1a, R1a or R1b).

Table S1: Sample sizes by FINRISK cohort and data type after quality control.

Table S2: Haplogroups Identified by YLineageTracker. Frequencies for haplogroups with at least 5 carriers are given. The "location in phylogeny " refers to which phylogenetic tree clade the carriers of a certain YLineageTracker haplogroup belong with the number in brackets indicating the number of samples. Haplogroup name in YFull and Karmin et al. (2015) are annotated using the YLineageTracker terminal markers.

Table S3: Phylogenetic relationships of variants within haplogroup N1a1 with at least 5 carriers. Annotations (ISOGG, YFull, Karmin et al. (2015), Ilumäe et al. (2016)) are based on all observed SNPs. Some annotations are contradicting due a variant having misclassification in a database, or due to the variant being recurrent.

Table S4: Phylogenetic relationships of variants within haplogroup I with at least 5 carriers. Annotations (ISOGG, YFull, Karmin et al. (2015)) are based on all observed SNPs. Some annotations are contradicting due a variant having misclassification in a database, or due to the variant being recurrent.

Table S5: Phylogenetic relationships of variants within haplogroup I with at least 5 carriers. Annotations (ISOGG, YFull, Karmin et al. (2015)) are based on all observed SNPs. Some annotations are contradicting due a variant having misclassification in a database, or due to the variant being recurrent.

Table S6: All haplogroup clades within the phylogenies of I, N, and R. Scaled frequency refers to normalized frequency out of all samples, and scaled out of major refers to normalized frequency out of major haplogroup carriers.

Table S7: TMRCA estimates for selected N1a1 haplogroups.

Table S8: Geographical enrichment test out of all samples. Significant refers to enrichment test $p<0.05$ and Significant multiple refers to $p<0.05/103$**.**

Table S9: Geographical enrichment test out of major haplogroup I1a, N1a1, R1a or R1b. Significant refers to enrichment test $p<0.05$ and Significant multiple refers to $p<0.05/96$**.**

Table S10: Pairwise-FST values (lower triangular) and their standard errors (SE) (upper triangular) between 19 Finnish regions and Estonia calculated for 23,434 FINRISK samples and 32 Estonian samples from Tambets et al. (2018). The FST values and their SEs have been multiplied with 100,000, and the color intensity corresponds to increasing FST value.

Figure S1

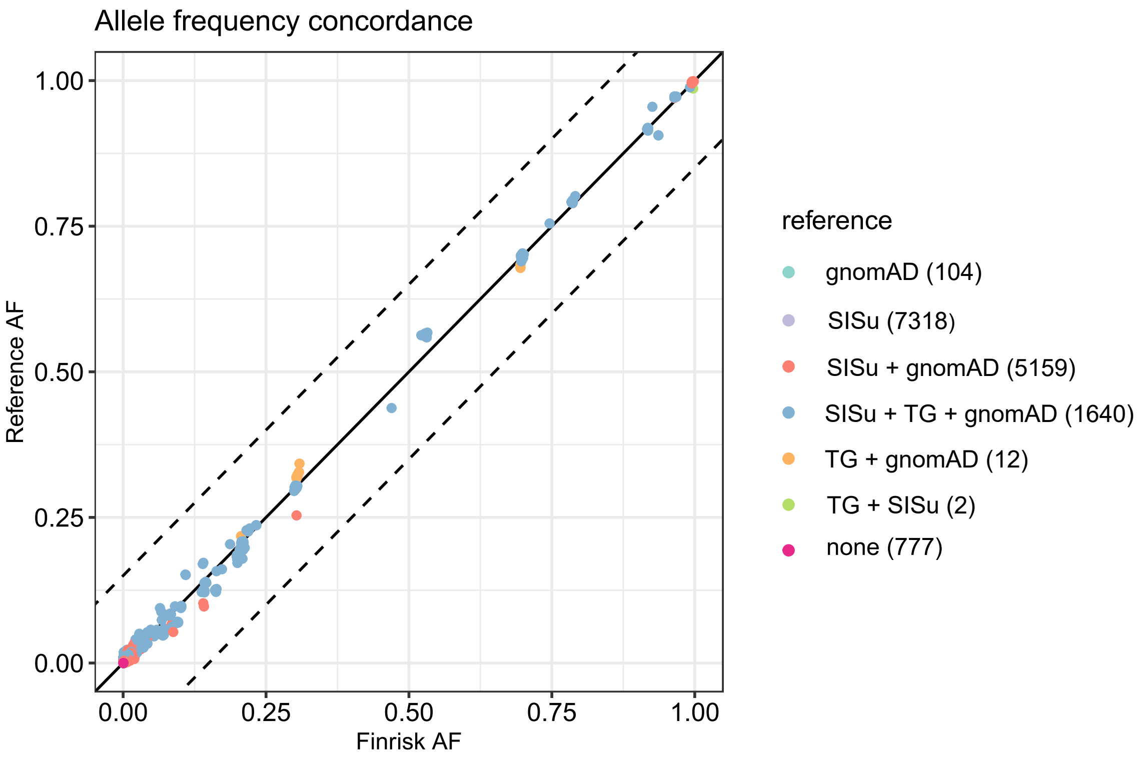

Figure S1 Allele frequency concordance with three Finnish WGS datasets: SISu v4, 1000 Genomes Project and Human Genome Diversity Project data Finns (TG), and gnomAD v3.1.2 Finns. We accepted the allele frequencies to differ up to 15 percentages between FINRISK and the reference data sets, since the variant frequencies are known to vary extensively based on the geographical location.

Figure S2

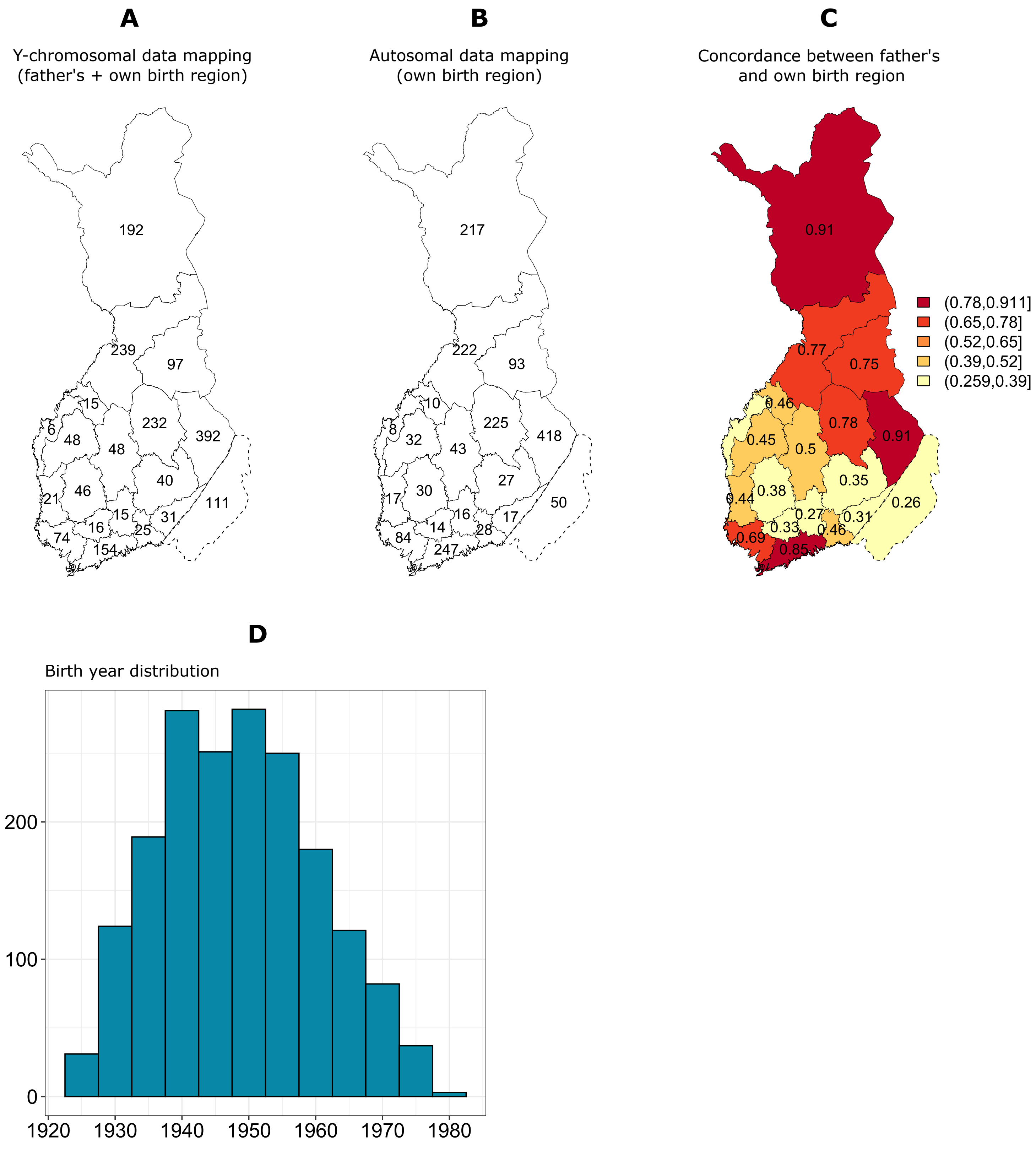

Figure S2 A) Sample distribution for Y-chromosomal analyses (N=1,802), and B) autosomal analyses (N=1,709). C) Concordance between father's and own birth region indicated as a proportion of fathers having their son born in the same region. D) Sample distribution by birth year.

Figure S3

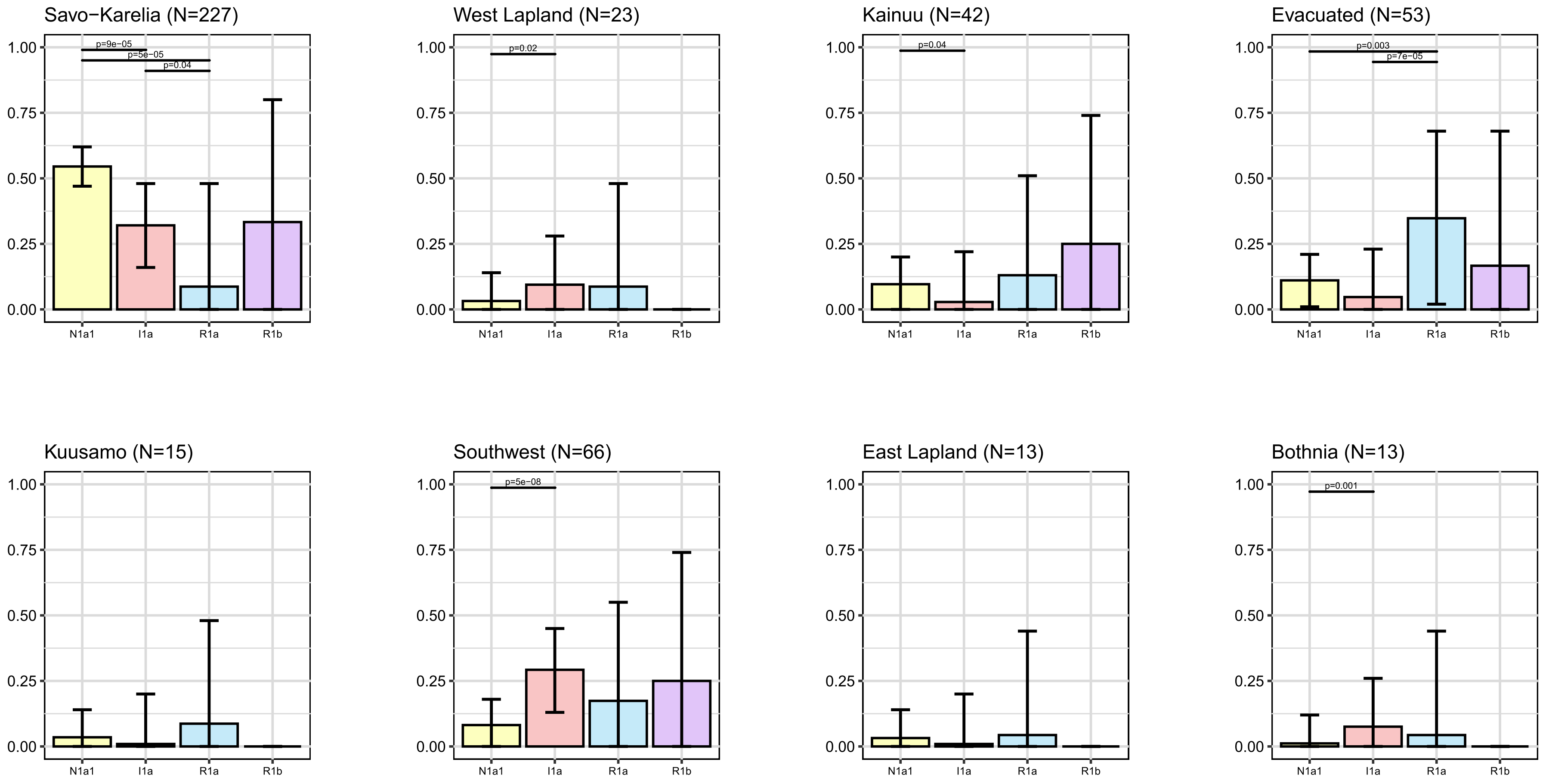

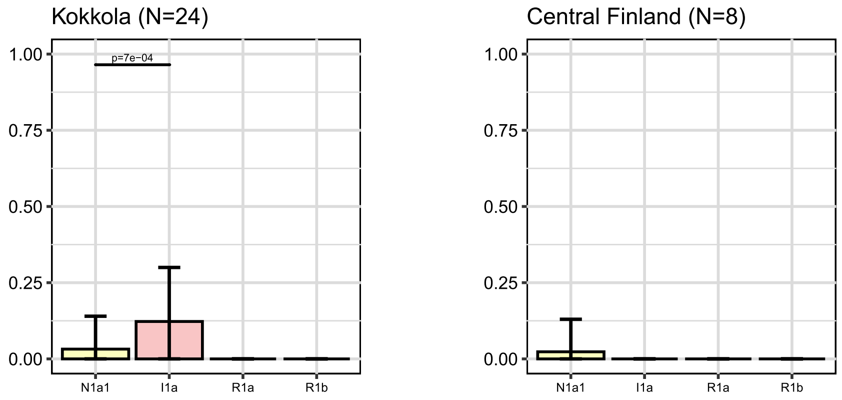

Figure S3: Autosomal ancestry proportions for major haplogroups N1a1, I1a, R1a, R1b. Significance testing is based on two-sample proportion test.

Figure S4

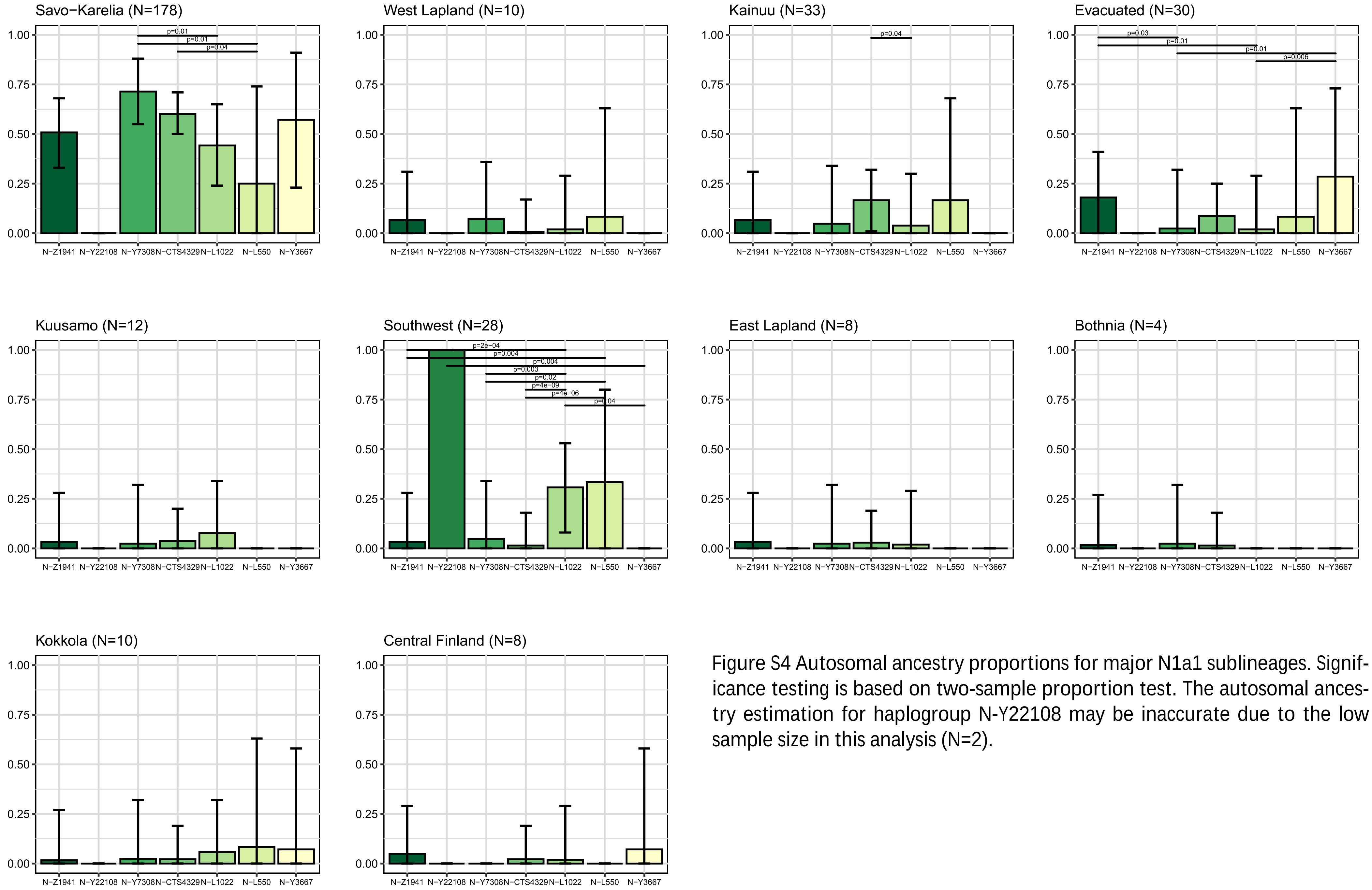

Figure S4 Autosomal ancestry proportions for major N1a1 sublineages. Significance testing is based on two-sample proportion test. The autosomal ancestry estimation for haplogroup N-Y22108 may be inaccurate due to the low sample size in this analysis (N=2).

Figure S5

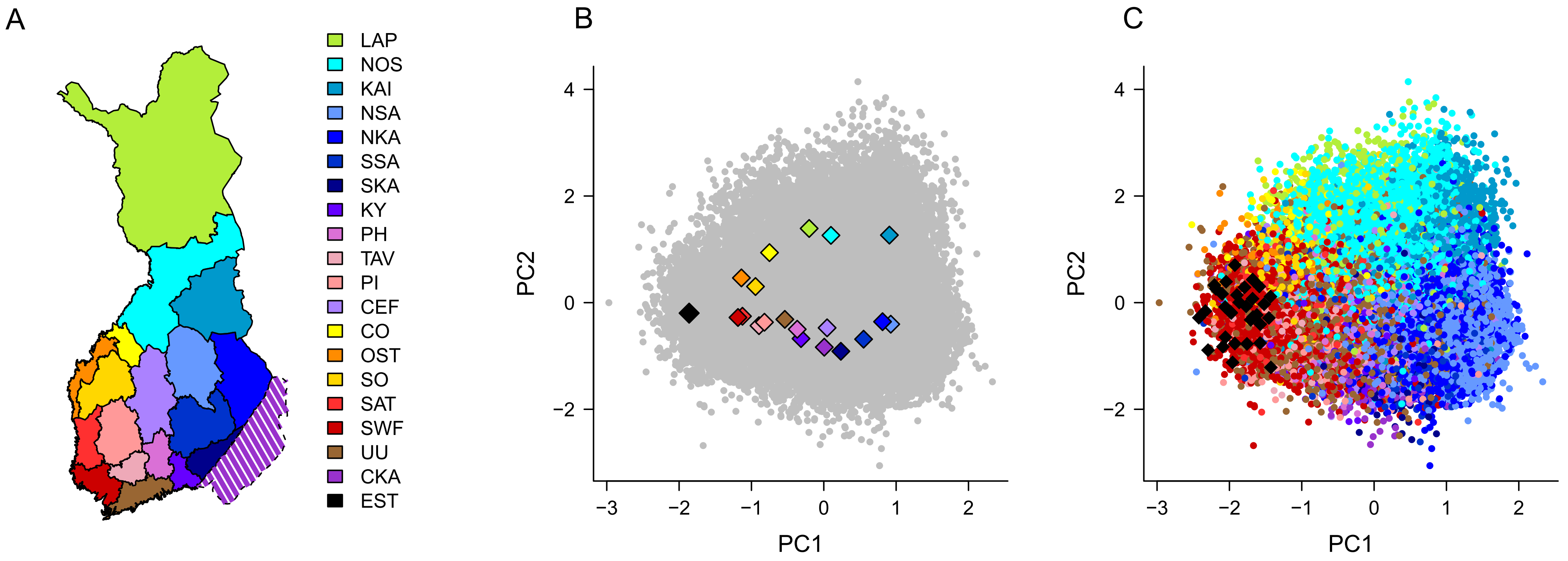

Figure S5 Genetic relatedness between Finns and Estonians. A) Geographical distribution of 23,434 FINRISK samples used for joint PCA analysis together with 32 Estonians from Tambets et al. (2018) B) PC1 vs. PC2 for the combined dataset of Finns and Estonians. The PCs are standardized (mean 0, variance 1). Gray dots represent individual samples and diamonds correspond to the mean PC score from each geographical location. C) PC1 vs. PC2 colored by each sample's geographical location. Estonian samples are visualized with black diamonds.
